# The impact of COVID-19 on fertility behaviour and intentions in a middle income country

**DOI:** 10.1371/journal.pone.0261509

**Published:** 2022-01-06

**Authors:** Tom Emery, Judith C. Koops

**Affiliations:** 1 Department of Public Administration, Erasmus University Rotterdam, Rotterdam, Netherlands; 2 Netherlands Interdisciplinary Demographic Institute (NIDI)-KNAW, University of Groningen, Groningen, Netherlands; University of Botswana, BOTSWANA

## Abstract

The COVID Pandemic may affect fertility behaviour and intentions in many ways. Restrictions on service provision reduce access to family planning services and increase fertility in the short term. By contrast, the economic uncertainty brought about by the pandemic and its impact on mental health and well-being may reduce fertility. These various pathways have been explored in the context of high income countries such as the United States and Western Europe, but little is known about middle income countries. In this paper we asses the impact of the COVID pandemic on fertility intentions and behaviour in the Republic of Moldova, a middle income country in Eastern Europe, using the Generations and Gender Survey. This survey was conducted partially before and partially after the onset of the pandemic in 2020, allowing for detailed comparisons of individual circumstances. The results indicate that the pandemic reduced the used of intrauterine devices, and increased the use of male condoms, but with no overall decrease in contraceptive use. Conversely individuals interviewed after the onset of the pandemic were 34.5% less likely to be trying to conceive, although medium term fertility intentions were unchanged. Indicators therefore suggest that in the medium term fertility intentions may not be affected by the pandemic but restricted access to contraception requiring medical consultation and a decrease in short-term fertility intentions could disrupt short term family planning.

## Introduction

It is expected that the COVID-19 pandemic will influence fertility levels [1]. Similar to previous crises—like the 2008 economic crisis—economic uncertainty can reduce the intention to have a child and therefore depress fertility behaviour. However, there is also something unique about the current crisis, namely, the pandemic’s possible impact on access to family planning. The most effective and common way to reduce fertility is with the use of contraceptives. However, due to the current health-crisis, access to family planning services may actually be reduced in some contexts. In addition, the COVID-19 pandemic is different because it restricts the movement of people, people are at home more, and this may influence family relations and sexual activity [2]. Tension between intentions on the one hand and actual behaviour on the other makes it unclear how the pandemic will affect the overall fertility level.

Most of the existing research on the impact of the pandemic on fertility behaviour has been concentrated on developed economies [3]. Aassve et al. (2020) argue that the impact of the pandemic on fertility will largely be shaped by the socio-economic context of individuals. They argue that in high income countries, damage to work-life balance and the access to assisted reproductive technology will negatively affect fertility. This will be amplified by the increase in economic uncertainty, which evidence from the great recession of 2008 suggests will depress fertility levels. By contrast rural areas of low-income countries could observe an increase in fertility as access to family planning is curtailed and general development hampered or even declined [4].

They see greater uncertainty about the impact on fertility in middle income countries and particularly urban areas within them. In such places, Aassve et al. (2020) argue that it is unclear if reduced access to family planning would lead to an increase in fertility and whether this would offset any decrease in fertility brought about by the uncertain economic outlook. The puzzle presented is a complex one with multiple influences on the fertility intention and behaviour of individuals. Attempts have been made to conceptualize this and produce more precise workable hypotheses but these again focus on high income countries [5]. Whilst fertility data is increasingly revealing the short-term impact of the pandemic on fertility, the aim here is to examine the role played by these competing causal pathways in shaping fertility behaviour at the individual level in a middle-income country such as the Republic of Moldova during the first months of the pandemic in 2020. In middle income countries it should also be noted that the impacts of the pandemic are likely to affect the population for longer given that vaccine role out is expected to be far slower in middle income countries than in high income countries.

## Existing research

### Fertility intentions

Research on the impact of the pandemic on fertility behaviour has been largely concentrated on high income countries, predominantly in Western Europe and North America. The evidence here has suggested a fall in fertility intentions in line with the hypothesis put forward by Aassve et al. (2020). Luppi, Arpino and Rosina (2020) demonstrated that fertility intentions had fallen in Spain, France, Italy, Germany and the UK but that this manifested itself differently in each context. In Italy, falls in fertility intentions were amongst the highly educated under 30’s whereas in Germany the patterns were geo-spatially concentrated in areas with the highest infection rates.

Research in Shanghai has demonstrated that fertility intentions amongst couples remain unaffected by the pandemic, especially if the couple themselves have faith in government and public health measures [6]. This is the only research to date that we were able to identify that wasn’t focused on a high income, western society. They also noted that those who intended to defer pregnancy plans were particularly worried about the impact of the virus itself on the health of the mother and fetus.

Wilde (2020) used data on google search terms to estimate that births would fall by 15% year on year by November 2020, reflecting a change in search term trends related to pregnancy and contraception [7]. They also noted that the fall was expected to be even higher amongst low income households and those from minority groups. This observation is consistent with understanding that economic uncertainties effect on fertility behaviour will be greatest amongst those with the fewest resources. However, the effect is very large and would represent a fall in fertility that is several times larger than that observed with the onset of the great depression in 2008.

### Sexual and reproductive health during the COVID-19 pandemic

COVID-19 and the associated social restrictions have raised concerns that access to family planning services could be restricted with significant consequences for family planning. The United Nations Population Division has suggested that the pandemic could result in 60 million fewer users of contraceptive methods worldwide [8]. The impact of the pandemic is expected to be particularly acute in low and middle income countries. Projected declines are expected to be largest for injectables (-20%), condoms (-10%) and the pill (-10%). The condom and the pill are pervasive in Eastern Europe and therefore the impact in this region could be particularly strong [9]. Furthermore, it is expected that rural populations access to family planning services will be disproportionately affected during the pandemic as supply lines and services are more thinly distributed [8]. It should be expected that contraceptive methods that require medical consultations will have particularly noticeable falls where as methods that can be used independently will be less affected.

Despite this there is currently little empirical data on the access to sexual and reproductive health services during the pandemic in low and middle income countries. The implicit assumption appears to be that access to family services in high income countries would be unaffected by the pandemic and associated restrictions on movement and businesses [3, 4]. The rationale for this belief is somewhat understandable as some forms of contraception are readily available. However, during the lockdown the office hours of doctors and various clinics were reduced, and services altered. Lindberg et al showed that around 28% of childbearing age in the United States worried about their access to sexual and reproductive health care due to the pandemic and 39% saying they had delayed or cancelled such care services due to the pandemic [10]. These concerns and limitations on access are greater amongst marginalized or vulnerable populations.

In addition to contraceptive use, a lockdown may lead to an increase in sexual activity [11]. However, several surveys conducted during the pandemic have shown this to not be the case and that instead sexual activity appears to have declined [12].

### The progression of the pandemic in the republic of Moldova

The aim of this paper is to examine the impact of the COVID pandemic on fertility in a middle income country. However, the analysis is limited to the Republic of Moldova which has several characteristics which make it atypical for middle income countries. The World Bank defines a middle income country as one in which GNP per capita is between $4,046 and $12,535. As of 2019, the Republic of Moldova is at the bottom end of this range at $4,320. Most of these countries also lie in Latin America, Asia and Africa, however the Republic of Moldova is a middle income country that borders the European Union and Ukraine.

The Republic of Moldova also has specific features which are of relevance to the pandemic’s impact. Due to the Republic of Moldova’s proximity to countries with significantly higher income levels and extensive free-trade and association agreements with those countries the degree of out-migration and seasonal work migration is exceptionally large in the Republic of Moldova. Estimates by the UNFPA suggest that around 9.9% of the working age population is abroad for work [13]). Population and migration estimates are currently under extensive revision. At the time of writing these figures were yet to be published but it is anticipated that the total population will be substantially lower. This also means that remittances make up almost 12.5% of Gross Domestic Product [14]. Such extensive migration has in itself been shown to be disruptive to fertility intentions and their realization. In addition, the Republic of Moldova already had a low total fertility rate of around 1.27 in 2019 and it has been at this level since the turn of the century. Due to extensive out migration and low fertility, the Republic of Moldova is a rapidly ageing society and its demographic resilience is a key policy concern for the Moldovan Government and especially regarding the impact of COVID-19 on wider socio-economic development.

**Fig 1:**
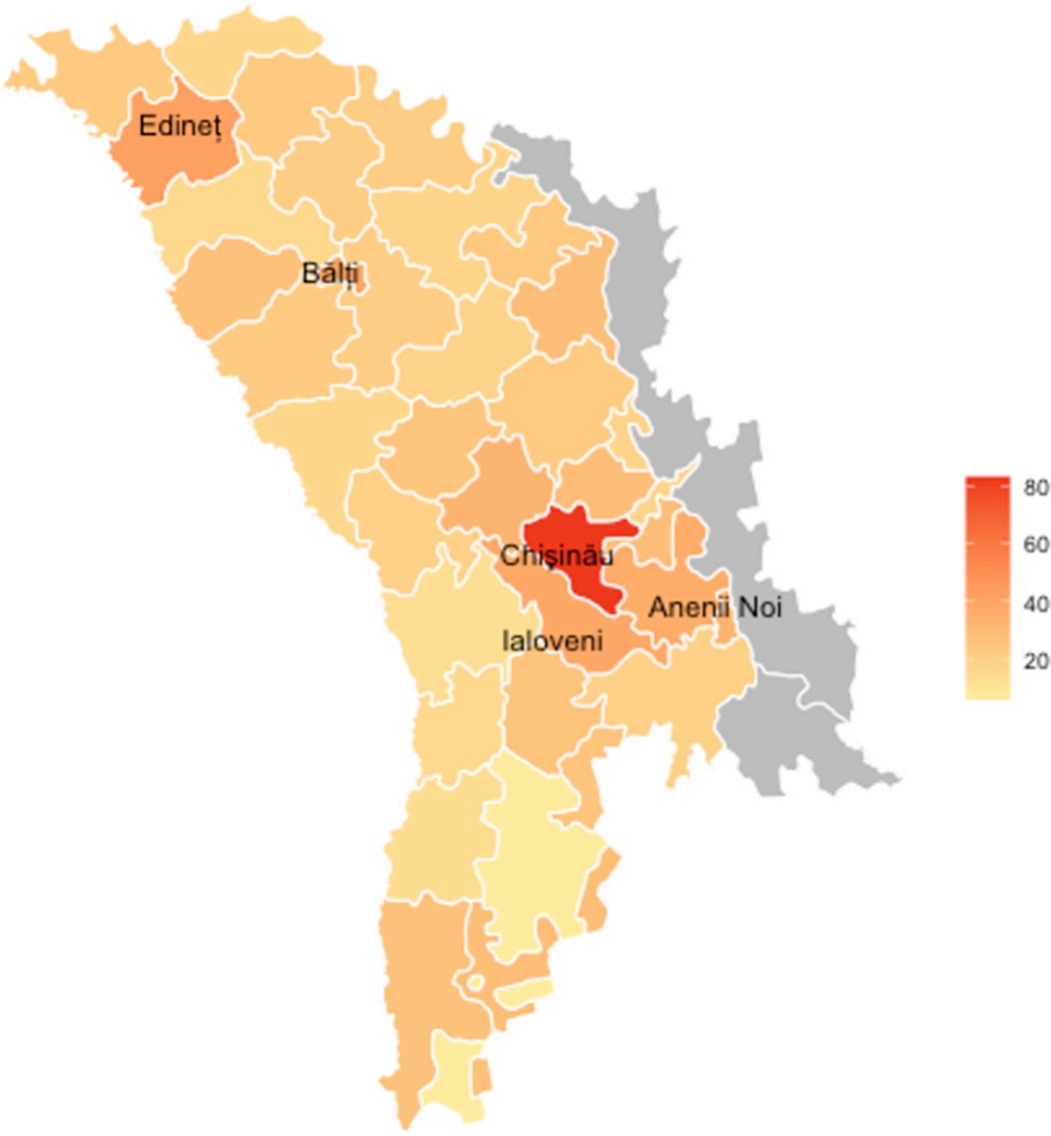
Cumulative cases of COVID-19 per thousand as of 21^st^ December 2020. Source—https://covid19.who.int/region/euro/country/md; Authors’ own illustration; Reprinted under a CC BY license, with permission from UNHCR, original copyright 2021: https://data.humdata.org/dataset/moldova-administrative-level-0-1-boundaries.

The progression of the pandemic in the Republic of Moldova was similar to that of many countries in Eastern Europe with low and moderate rates of infection relative to Western Europe in the early phase of the pandemic through March and April. However, there was a steady rise in cases through the summer and autumn months. The spread of the virus through the country has not been even. Anenii Noi, Bǎlți, Edine t and Ialoveni had the highest cumulative number of cases per thousand in the country peaking at around 35-40, with the capital Chișinău recording a rate of 83 (see Fig 1). Rural areas had much lower case rates than these urban centres.

**Fig 2:**
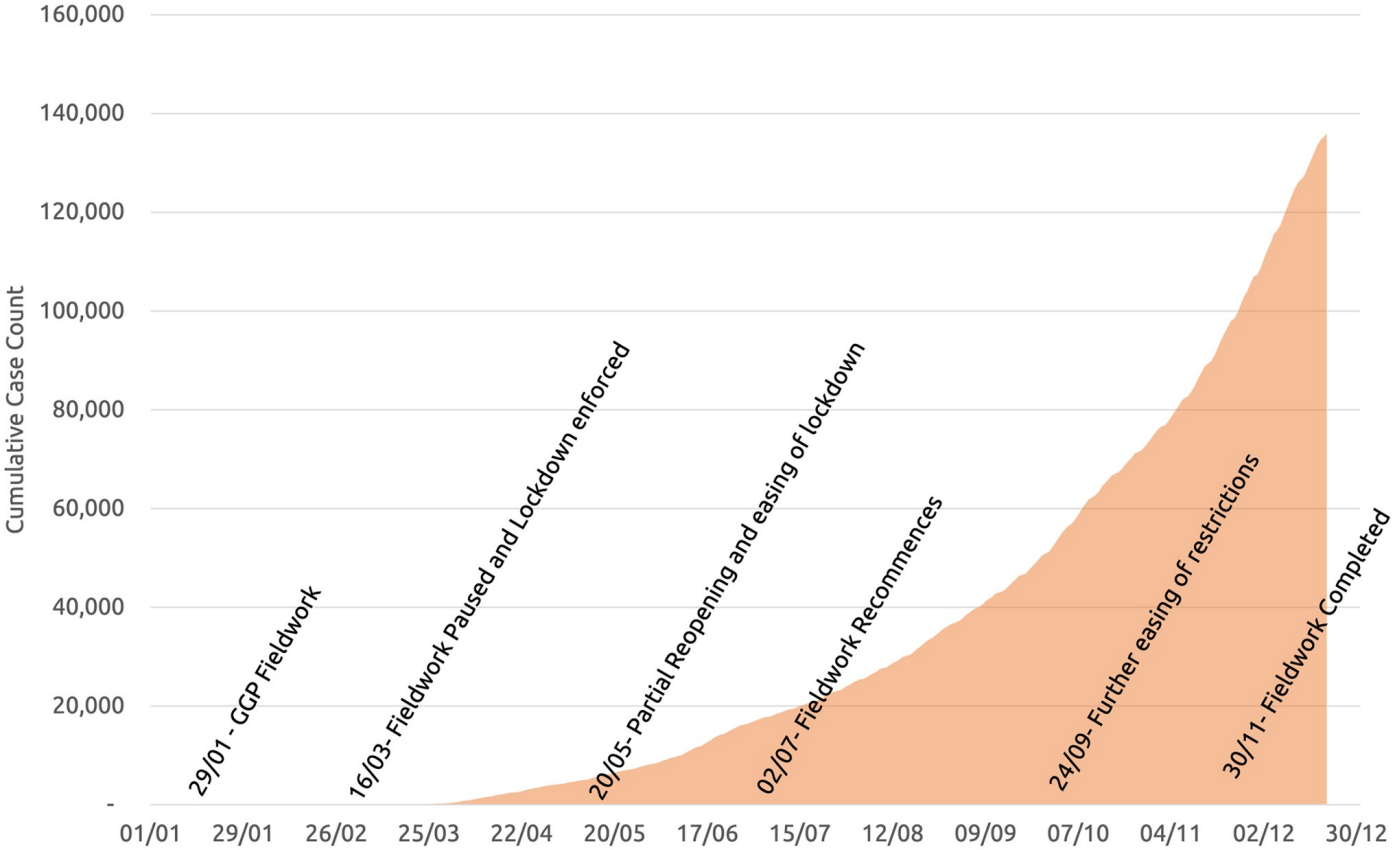
Cumulative cases of COVID-19 and timeline of events [16]; Authors’ own vizualization and annotations.

With regards to the regulations put in place, the Republic of Moldova was under an extensive lockdown from March through mid-May (see Fig 2). All schools were closed, as were most places of work. From mid-May, regulations were relaxed to allow recommencement of some activities as long as they allowed for adequate physical distancing of 1.5 meters. This allowed for the partial reopening of schools before the summer break though reduced class times or rotational timetables were used to ensure compliance with regulations. As of 24^th^ September, the distance required between individuals was reduced to 1 meter, allowing for schools to reopen more extensively and a further normalization of activities. These restrictions were in place through to the end of fieldwork.

With regards to access to modern contraceptive methods in the Republic of Moldova, existing research shows that use is far less common than in Western Europe but that socio-economic differences in contraceptive use are not identifiable [15]. During the strictest parts of the lockdown non-emergency medical consultations were postponed which may suggest a shift away from methods requiring medical consultation towards more widely available contraceptive methods. It is very unclear however as to whether the lockdown itself has increased socio-economic inequalities in access to contraceptive methods.

Travel restrictions required the quarantining of individuals arriving from other countries. This is of particular importance in the context of the Republic of Moldova given the large rates of out migration and remittances. Travel restrictions to neighbouring countries therefore represented a severe disruption to economic and family activities.

### Reasoned action approach & hypotheses

To address the research question at hand, we adopt a reasoned action approach [17] to study changes in capacities, intentions and behaviour over the course of the pandemic. The reasoned action approach argues that intentions and behaviour are shaped by individuals perceived behavioural control. These intentions then shape behaviour and this is moderated by the barriers and facilitators of the planned behaviour. This approach is particularly useful in this context as it clearly delineates between shifting perceptions of the individual and external exogenous influences on behaviour. This helps address the question of whether the primary influences of the pandemic on fertility are associated with its impact on the perceptions of individuals or concentrated on the practical and logistical constraints that the pandemic imposes on individuals. This is a crucial distinction as recent research has shown that perceived barriers and uncertainty can have a significant impact on behaviour, irrespective of whether the barrier is actually realized [18]. For these reasons the approach has been valuable and widely used in the fertility literature and was the basis for the design of the Generations and Gender Survey [19].

By applying the reasoned action approach to data on the Republic of Moldova over the onset of the pandemic in 2020, this paper aims to not only estimate the change in intentions and behaviour brought about by the pandemic but also the degree to which these changes are associated with a shift in norms, perceived behavioural control or actual behavioural control. In doing so it will provide empirical insights to the puzzle presented by Aassve et al. (2020) regarding the impact of the pandemic on middle income countries such as the Republic of Moldova.

Based on existing empirical findings, the reasoned action approach, and the specific Moldovan context we derive the following hypotheses with regards to changes in fertility intentions and behaviour over the course of the pandemic:

*H1: Sexual Activity will be lower after the onset of the pandemic than before*.
  Existing research on mental well being and sexual relations during the pandemic has tended to show a decline in sexual activity during the pandemic, although these have not been as well studied in middle income countries [12].
*H2: Contraceptive use will be lower after the onset of the pandemic*
  Existing research and theories regarding the impact of the pandemic have generally suggested that access to contraception will decline but that this decline will be acutest for those methods which require family planning services, at least at the initial stage of starting a contraception plan [10].
*H3: The decrease in the use of contraceptives will be greatest in rural areas*
  One of the key distinctions made by Aassve et al. (2020) in their prediction of the fertility impact of COVID is between rural and urban areas and this relates in part to the potential differential impact on access to family planning services in rural areas during the pandemic.
*H4: Respondents will be less likely to be actively trying to get pregnant after the onset of the pandemic than before*.
  In addition to a drop in sexual activity and access to family planning services, it is also expected that there will be a decline in the proportion of couples who are actively trying to conceive. This is likely due to economic uncertainty and potential wider mental health impacts.
*H5: Fertility intentions will be lower after the onset of the pandemic than before*.
  Finally, it is expected that due to the aforementioned shifts in access to family planning services, changes in sexual activity as well as negative impacts of economic uncertainty, the fertility intentions in the Republic of Moldova to fall.
*H6: Fertility intentions will decline most amongst households who have lost income due to the pandemic*.
  In addition to a differential impact through access to contraception, existing research on exogenous shocks and fertility have demonstrated that households whose income is directly hit by the shock exhibit the biggest drops in fertility intentions and this has been supported by existing evidence on the impact of COVID-19 [18]. We build upon this research by estimating the effect of personal loss of income due to the pandemic on fertility intentions. In addition, we examine whether low socio-economic status in and of itself can explain differences in intentions.

## Data & methods

### Data

This paper uses data from GGS Wave 1 (DOIs: 10.17026/dans-z5z-xn8g), see Gauthier, A. H. et al. (2018) or visit the GGP website (https://www.ggp-i.org/) for methodological details. The GGS is cross-national longitudinal survey of family and relationship dynamics that is currently undertaking a new round of data collection. The questionnaire was designed by an international team of demographers through the Generations and Gender Progamme (GGP) [20]. This questionnaire was then translated into Romanian and Russian and validated by the UNFPA and GGP. In the Republic of Moldova the fieldwork was overseen by the UNFPA in coordination with the National Bureau of Statistics of the Republic of Moldova. Informed oral consent was attained from all participants in compliance with local statistical regulations and the General Data Protection Regulation (Regulation (EU) 2016/679). The target sample was the residential population aged 15-79. All interviews were conducted as face-to-face. Around 15% of the area and population of the Republic of Moldova are made up of a break away region called Transnistria which is defacto independent of the state. This regions was not covered in the data collection.

Fieldwork commenced on the 29^th^ January 2020. On the 16^th^ March fieldwork was paused indefinitely due to physical distancing measures put in place across the Republic of Moldova. Fieldwork recommenced on the 2^nd^ July and was then conducted continuously until 30^th^ November 2020. This resulted in 10,044 interviews. In the context of the actual prevalence of the disease, the study covers a period in which the number of cases were increasing. 3,055 interviews were conducted before the onset of the pandemic and a further 6,989 interviews were conducted afterwards. More information about the fieldwork procedures can be found on the GGP website and accompanying documentation (https://www.ggp-i.org/data/methodology/) and further information on the Moldovan fieldwork can be found on the Online Data Browser (https://www.ggp-i.org/data/browse-the-data/).

The overall response rate was 62.9%. Response rates in Chiinău were much lower than the rest of the country at 41.6% reflecting a trend that is well known for surveys in Eastern Europe [21]. This was despite extensive measures being undertaken to improve the response rate in Chiinău. The overall item non-response rate was very low at 3.9% and even lower in the fertility section at 0.66% which includes several highly sensitive questions, many of which are used in this analysis.

After the survey was interrupted due to COVID, the UNFPA and Moldovan National team requested that several items be added to measure the subjective experience of the pandemic and whether respondents felt that the pandemic had negatively affected various aspects of their lives. These questions were placed at the end of the interview to avoid effecting the core questionnaire.

### Variables

The hypotheses set out above refer to four separate dependent variables. Sexual activity is measured using question FER13 (Did you have sexual intercourse in the past 4 weeks? Yes or No). Contraceptive usage is measured using FER12 (Are you or your partner using or doing any of these things to prevent pregnancy at this time? Please name all of the things you use or do. Condom, Pills, Intrauterine Device (IUD), Diaphragm, Foam/Cream/Jelly/Suppository, Injectables, Implants, Pesona, Morning after pill, Withdrawal, safe period method, vaginal ring, female condom.) Those who selected a modern contraceptive method (Condom, Pills, IUD, Diaphragm, Foam/Cream/Jelly/Suppository, Injectables, Implants, Morning after pill, vaginal ring, female condom) were coded as 1 to reflect contraceptive usage (CU) and those who didn’t were coded as 0.

Traditional methods of contraception such as withdrawl and the rhythm method were also asked about but these were omitted from the analysis. Between the pre and post lockdown proportions of the fieldwork there was a small change in the way the question was presented that meant respondents post lockdown were offered a ‘none of the above’ option whilst those prior to lockdown were not. The inclusion of this response option seems to have drastically decreased the proportion of individuals reporting the withdrawal method. This in itself is interesting as it may suggest that the reporting of such traditional methods is highly sensitive to non-response options. Given this, the traditional methods were excluded from the analysis.

To measure whether a couple is actively trying to get pregnant FER10a was used (Are you or your current partner trying to get pregnant? Yes or No”). The dependent variables are presented in Table 1.

**Table 1:** Dependent variables.

	(1) Pre Lockdown		(2) Post Lockdown	
	mean	sd	mean	sd
Had intercourse	0.852	0.356	0.886	0.318
Contraceptive Use	0.389	0.488	0.408	0.492
Trying to Conceive	0.087	0.282	0.059	0.235
Fertility Intention	0.331	0.471	0.350	0.477
Observations	734		1909	

**Fig 3:**
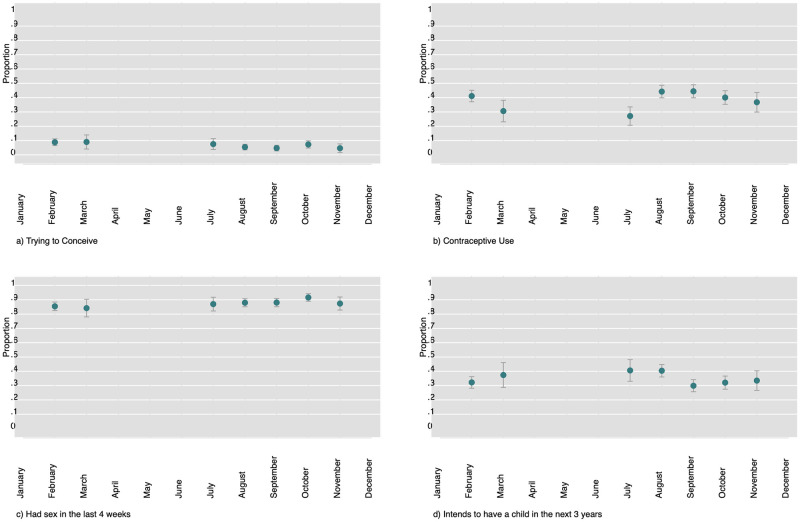
Proportion of 18-49 year old’s that are currently trying to conceive, using modern contraceptive methods, that have had sex in the last four weeks and that have fertility intentions in the next 3 years.

To measure a respondents fertility intention FER14 was used (Do you intend to have a/another child during the next three years? Please take into account only biological children. Definitely yes, probably yes, unsure, probably no, definitely no.”). It should be noted that the questions are only asked to women aged 18-49 and men who have a female partner aged 15-49. If the respondent or their partner were pregnant at the time of the interview were also excluded from the analysis. If they were not sure yet, they were included in the analysis. Those without a co-resident partner were asked these questions but we restricted the analysis to those with a partner and aged 18-49 in order to focus the analysis on those individuals most likely to have medium term fertility intentions. The changes in these four dependent variables over the course of the fieldwork is visible in Fig 3.

Independent variables of interest that are used in the analysis are age, sex, educational level (measured as whether an individual has tertiary qualifications or not), employment status (whether someone is in paid employment or on leave from paid employment), region, whether they live in an urban or rural area, and whether they have co-resident children (see Table 2). Questions on the impact of the pandemic on household income were only asked to the sample interviewed after the onset of the pandemic. For this analysis, those who responded that there was a large or very large impact on their household income were coded as 1 and the rest, including those who were interviewed before the onset of the pandemic, were coded as 0. The summary all the variables used can be found below:

**Table 2:** Independent variables.

	(1) Pre Lockdown		(2) Post Lockdown	
	mean	sd	mean	sd
Age	36.413	7.677	35.819	7.618
Sex of Respondent [Ref = Female]	0.357	0.479	0.327	0.469
Education Level	0.337	0.473	0.361	0.481
Employment Status	0.658	0.475	0.654	0.476
Number of Coresident Children	1.598	1.145	1.553	1.044
Urban Resident	0.475	0.500	0.335	0.472
Willingness to answer	0.655	0.476	0.653	0.476
Observations	734		1909	

### Analytic strategy

For the analysis, the hypotheses are tested in turn using a logit model, using a dichotomous variable to represent the period before and after the onset of the pandemic. However, the onset of the pandemic has itself been made up of several stages in the Republic of Moldova. To test the findings in these models, they were also run with time specified as a categorical month variable to allow for variation in the impact of the pandemic over time. Generally, the effects were stable over the months preceding the resumption of fieldwork and so the simpler pre and post measure is used but where monthly variations are statistically significant, they are reported.

Post stratification weights were applied based on age, gender and region of residence. The primary internal validity concern relates to the progression of fieldwork in the Republic of Moldova and its association with the progression of the pandemic. In survey research it is common to observe that specific groups respond earlier in the fieldwork period than others. It is likely that these different groups will have different fertility intentions and behaviours and this thus threatens to bias the estimates. It is likely that those which are hardest to reach are those more likely to migrate or be less settled and these are also those individuals least likely to be intending to have children. However, the fieldwork in the Republic of Moldova was structured such that various districts of the sample were exploited at different times and were exhausted before the fieldwork team moved on to the next district. The onset of the pandemic coincided with the completion of the first round of districts which may go someway to mitigating this effect. The districts examined in each round of fieldwork were approximately representative of the country as a whole.

However, to further test against this potential bias, models were run which included variables indicating respondent willingness to participate and the number of others present during the interview. These variables are recorded at the end of the interview by interviewers and are not seen by respondents. The willingness to answer questions is a standard quality control item in the GGS and is marked on a scale of 1-10. Physical distancing measures meant that some interviews were conducted outdoors or in larger rooms and there was therefore potentially less privacy in fieldwork after the onset of the pandemic, leading to increased social desirability bias.

Whilst these variables did exhibit a correlation with fertility behaviours and intentions, their inclusion in the models didn’t not change the significance or direction of the coefficients reported here. There was no statically significant difference in ‘willingness to answer questions’ pre and post pandemic (see Table 2) and this was also true of other data quality measures such as item non-response, length of interview and interview interruptions. The migration characteristics of respondents and their partners in the pre and post lockdown populations were also compared and no statistically significant differences were found, suggesting that there was not increased inward or outward migration associated with the lockdown which could account for differences in fertility behaviours.

## Results

The first set of results are presented in Table 3. Model 1 indicates that the odds of respondents reporting having had sex after the lockdown were 38% (*exp*(0.326) = 1.38) greater than the odds of respondents having had sex prior to lockdown and this is significant at *p* < 0.01. This runs counter to the first hypotheses and suggests that there has in fact been an increase in sexual activity after lockdown. When looking at the basic descriptive statistics by gender, the difference between pre and post lockdown is larger amongst women (82% v 86%) than men (92% v 94%).

**Table 3:** Results of logistic regression on pre & post population [Log Odds].

	(1) Had sex	(2) CU	(3) CU	(4) Trying	(5) Intention	(6) Intention
Post Lockdown	0.326**	0.138	0.0515	-0.608***	0.00208	-0.0359
	(2.58)	(1.66)	(0.46)	(-3.98)	(0.02)	(-0.32)
Age	-0.0129	-0.0240***	-0.0237***	-0.0267**	-0.117***	-0.117***
	(-1.65)	(-4.77)	(-4.72)	(-2.91)	(-18.53)	(-18.54)
Sex of Respondent [Ref = Female]	1.175***	-0.218**	-0.215**	0.0985	0.714***	0.715***
	(8.83)	(-2.90)	(-2.85)	(0.68)	(8.24)	(8.25)
Number of Coresident Children	-0.0799	0.0421	0.0431	-0.674***	-0.572***	-0.573***
	(-1.53)	(1.18)	(1.21)	(-8.48)	(-12.59)	(-12.60)
Higher Education [Ref = No]	0.241	0.382***	0.376***	-0.0467	-0.0204	-0.0213
	(1.77)	(4.58)	(4.49)	(-0.29)	(-0.21)	(-0.22)
Working	0.385**	0.187*	0.185*	0.103	-0.0222	-0.0180
	(3.21)	(2.27)	(2.25)	(0.64)	(-0.24)	(-0.19)
Urban	0.164	0.0316	-0.0994	0.0231	0.272**	0.270**
	(1.23)	(0.38)	(-0.71)	(0.15)	(2.87)	(2.85)
Willingness to answer [1-10]	0.0591	-0.407***	-0.411***	0.203	0.00303	0.00806
	(0.49)	(-5.26)	(-5.30)	(1.29)	(0.03)	(0.09)
Others Present	0.0783	0.377***	0.377***	-0.350	-0.0770	-0.0762
	(0.48)	(3.81)	(3.82)	(-1.59)	(-0.67)	(-0.67)
Post Lockdown X Urban			0.194			
			(1.16)			
Drop in Income = 1						0.0661
						(0.66)
Post Lockdown X Drop in Income = 1						0
						(.)
Constant	1.611***	0.263	0.323	-0.597	3.979***	3.978***
	(4.70)	(1.19)	(1.42)	(-1.48)	(15.01)	(15.01)
Observations	2289	2230	2230	2220	2114	2114

*t* statistics in parentheses

* *p* < 0.05,

** *p* < 0.01,

*** *p* < 0.001

The second model indicates that the odds ratio of using modern contraceptives was not statistically different after the lockdown. This is counter to the second hypothesis and the assertion by Aassve et al. (2020) that contraceptive usage would be constrained. The third model tested whether there was a difference in the impact of the lockdown between urban and rural areas but again there was no difference identified. In further analyses (not shown), interaction terms were also included between education level and the impact of lockdown and no significant effects or improvement in model fit were found. This suggests that the stability in the use of modern contraceptive methods was relatively stable across geo-spatial and socio-economic groups.

It is also of interest whether the stability in contraceptive use is constant across those who have medium term fertility intentions and those who don’t. In the terms of the United Nations definition of Sustainable Development Indicator 3.7.1, this is the distinction between an unmet family planning need associated with limiting or spacing of children. Amongst those with medium term fertility intentions, usage of contraception rose from 37% pre lockdown to 39%. Amongst those without any medium term fertility intentions, it rose from 43% to 45%. Neither difference is statistically significant so there is no evidence of substantial increases in the unmet needs with regards to either limiting and spacing of children.

**Fig 4:**
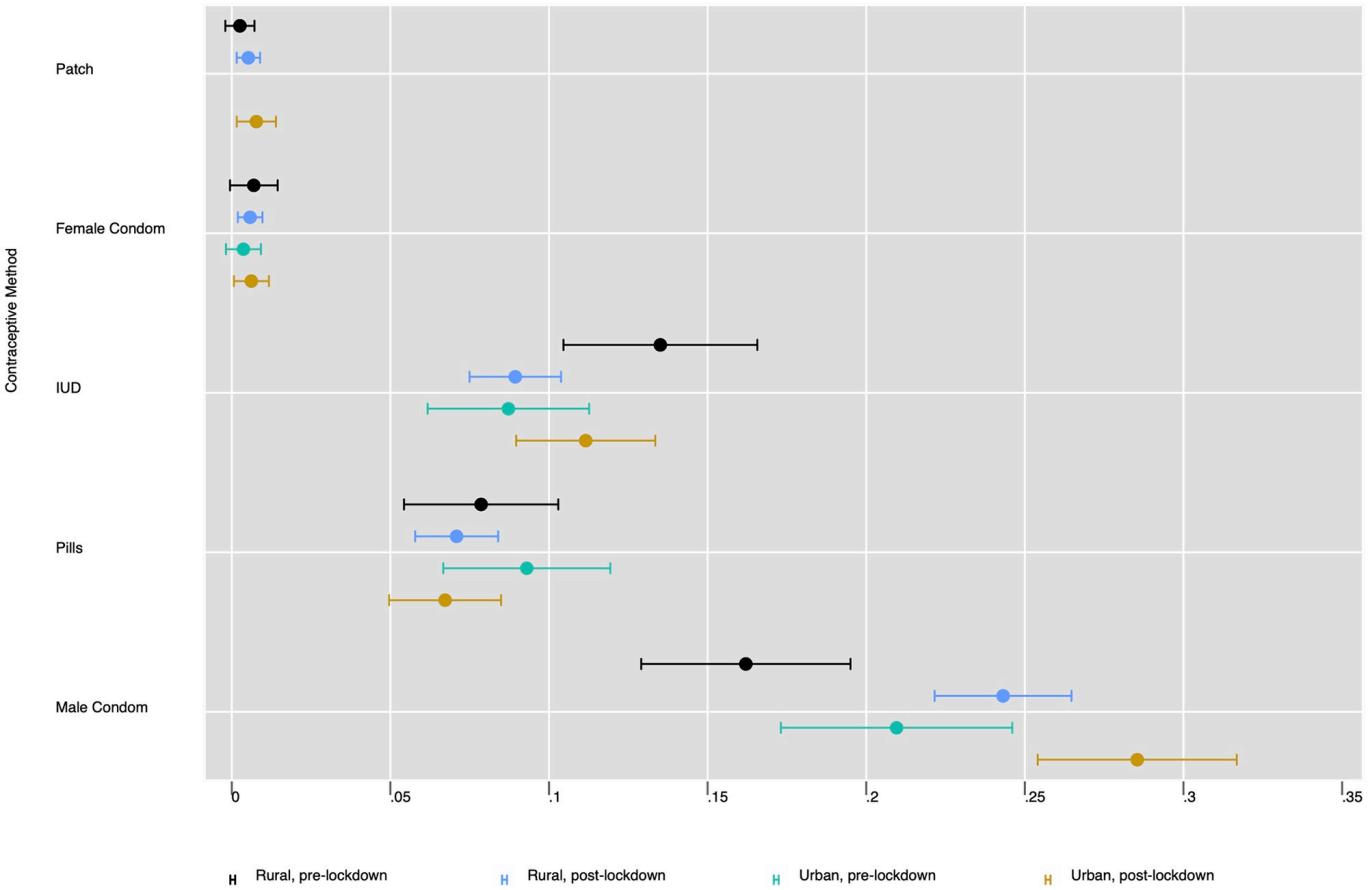
Marginal effects at the mean of contraceptive use pre and post lockdown.

As suggested by the literature, changes in contraceptive use were then analyzed by specific modern contraceptive method. For this we looked at the five methods that are pervasively used in Moldova: male condom, female condom, pills, IUD and patches. Model 3 was replicated the analysis for each individual method. The marginal effects at the mean for urban and rural populations, pre and post lockdown were then calculated and are presented in Fig 4.

Again the results indicate a high degree of stability in the use of modern contraceptive methods pre and post lockdown. However, there are two notable exceptions where statistically significant differences were observed. IUD use in rural areas fell 36% from 14% to 9%. Conversely male condom use in rural areas rose between the two time periods rose 21% from 19% to 23%, effectively offsetting the use of IUD’s. That this is only observed in rural areas is in line with the hypotheses of Aassve et al. (2020). Furthermore, the shift is from a method requiring a medical consultation (IUD) to one that does not (male condom) [8]. However, it should be noted that an IUD also needs a medical consultation at the end of use, so we are cautious about interpreting this as restriction on access to medical services.

The fourth model shows that the odds ratio of a respondent trying to conceive were 46% (1 − *exp*(−0.608) = 0.544) lower post lockdown than prior, with the reported rate dropping from 8.7% to 5.7%. That is a 34.5% drop in those actively trying to conceive and it supports the fourth hypothesis. The fifth and sixth models reveal no differences in the medium term fertility intentions of respondents between pre and post lockdown and there was no difference identified between those who were financially impacted by the lockdown and those who weren’t.

In terms of the robustness of the results, the potentially confounding factors are associated with the fieldwork process itself. Respondents in the second part of the fieldwork may have been more reluctant respondents. We do note that the perceived willingness to answer questions was associated with lower levels of contraceptive use, which is to be expected from a non-cooperative respondent as they may want to avoid providing details of a specific contraceptive method. This is unlikely to explain the observed effect of lockdown on contraceptive usage however, as the difference in means between pre and post lockdown is small, statistically insignificant and in the opposite direction from that of the effect of lockdown on contraceptive usage (see Table 2). To ensure that a differential in ‘willingness to answer’ was not driving this effect an interaction effect was included with the lockdown variable but the coefficient was not significant (results not shown).

We also considered the role of others being present during an interview. This was included because there was a change in fieldwork protocols after lockdown which may have influenced responses as it was harder to hold interviews alone. An association of others being present with the dependent variables was only apparent for contraceptive usage in models 2 and 3 but interestingly, those with others present reported higher levels of contraceptive usage which is contrary to expectations. It was expected that people would be shy about declaring specific contraceptive methods but the results suggest that the social desirability bias may operate the other way, with respondents more likely to declare to use a contraceptive method if others are present. This could suggest that the drop in contraceptive usage after lockdown is larger than reported.

## Conclusion

This paper seeks to understand the impact of the COVID-19 Pandemic and associated social measures on fertility behaviour in the Republic of Moldova. 75% of the world’s population live in middle income countries such as the Republic of Moldova. According to the world bank, and as laid out by Aassve et al., the potential impact of the pandemic on fertility behaviour in these countries is hard to anticipate. This paper offers some insights into the complex impact the pandemic has had on fertility behaviour but ultimately reinforces the assertion made by Aasvve et al. that the long-term demographic impact is hard to ascertain.

In terms of the overall impact on fertility there are several notable findings from this paper. Firstly, we observe a dramatic fall in the proportion of respondents trying to conceive of around 34.5%. If this was reflected in a similar fall in the birth rate, the demographic consequences of the pandemic would be considerable. However, the medium term fertility intentions do hold steady in our analyses. Given that existing research indicates that there is strong association between short term fertility intentions and behaviour [22], this might result in a delay in births rather than a permanent decline in the birth rate. However, the extent to which deferred conceptions will indeed be realized goes beyond the scope of this paper as this can only be established in a few years from now.

The other factor that Aassve et al. point to which complicates the picture in middle income countries, is the access to family planning services. We found some evidence that the lockdown resulted in restricted access to some specific contraceptive methods but these were largely offset by a switch to more readily available methods. Existing literature has suggested that there would be a fall in sexual activity associated with the socio-economic impact of the pandemic but this was not observed. Given this, we would not expect a significant increase in unplanned births and we therefore do not expect unplanned births to offset the fall in conception efforts amongst those with medium term fertility intentions.

There are several limitations we can point to in this paper. Most importantly, the analyses are based on a repeated cross-sectional study. The differences we observe are between and not within individuals. A longitudinal study would offer far greater insights into the shifts in fertility behaviour and the underlying dynamics. Unfortunately this was not possible with the data at hand. There are longitudinal panels which offer such data in high income countries (For example see Alburez-Gutierrez et al [23]). However, We are unaware of any such longitudinal data for middle income countries and the impact of the pandemic on fertility in such a context was the main focus of this paper. This will be particularly important when considering the medium and long term impacts of the pandemic as it will alter all manner of social processes such as partnership formation, educational attainment, labour market trajectories and many others. Understanding the impact of these processes on fertility behaviours in the aftermath of the pandemic is vital but this analysis only considers the short term impact on fertility intentions.

The second limitation is the focus of the analysis purely on survey data. Survey data provides subjective measures and insights into cognitive processes that are exceptionally valuable for fertility research. However, there have been serious developments in the use of new forms of data in the study of demography, such as google trends data, that could prove useful in validating the results found here [7, 23]. Doing so lies outside the scope of this paper given that the theoretical framework is deeply embedded within the survey data used and because the analysis of these new data forms require sufficient detail and attention that this paper can not accommodate.

Finally, the study focuses on the Republic of Moldova as an example of a middle income country. The Republic of Moldova is however an atypical middle income country country. Unlike most middle income countries it is in Europe and has a land border and association agreement with the European Union which have a significant gravitational impact on the economy and wider society. Whilst the Republic of Moldova does exhibit poor access to family planning in line with many middle income countries, it does not exhibit some of the crucial challenges that Aassve et al. referred to. The urban-rural divide in access to family planning in the Republic of Moldova may not be so great given that the country is relatively small in both area and population when compared with more notable middle income countries such as China, India, Indonesia or Brazil.

This paper has provided first insights into the association of the COVID-19 pandemic on fertility behaviour at the micro-level in the context of a middle income country. The impact of the pandemic on middle income countries is understudied and yet of critical importance in understanding the wider demographic implications of the pandemic. The data used has enabled us to look at multiple dimensions of fertility behaviour and how they interrelate. There is strong evidence that the pandemic led to a large reduction in short-term fertility intention and in the number of people actively trying to conceive. It will be vital that this is continuously studied as time passes.
